# Prebiotic Pathway from Ribose to RNA Formation

**DOI:** 10.3390/ijms22083857

**Published:** 2021-04-08

**Authors:** Gaspar Banfalvi

**Affiliations:** Department of Molecular Biotechnology and Microbiology, University of Debrecen, 1 Egyetem Square, 4010 Debrecen, Hungary; bgaspar@unideb.hu; Tel.: +36-52-512-900 (ext. 62319); Fax: +36-52-512-925

**Keywords:** ribose formation, fitting of aldopentoses, preRNA synthesis, hydrolysis of preRNA, phosphorylation of NMPs, genRNA synthesis

## Abstract

At the focus of abiotic chemical reactions is the synthesis of ribose. No satisfactory explanation was provided as to the missing link between the prebiotic synthesis of ribose and prebiotic RNA (preRNA). Hydrogen cyanide (HCN) is assumed to have been the principal precursor in the prebiotic formation of aldopentoses in the formose reaction and in the synthesis of ribose. Ribose as the best fitting aldopentose became the exclusive sugar component of RNA. The elevated yield of ribose synthesis at higher temperatures and its protection from decomposition could have driven the polymerization of the ribose-phosphate backbone and the coupling of nucleobases to the backbone. RNA could have come into being without the involvement of nucleotide precursors. The first nucleoside monophosphate is likely to have appeared upon the hydrolysis of preRNA contributed by the presence of reactive 2′-OH moieties in the preRNA chain. As a result of phosphorylation, nucleoside monophosphates became nucleoside triphosphates, substrates for the selective synthesis of genRNA.

## 1. Introduction

Fossilized microorganisms found in hydrothermal vents could have appeared on Earth around 4.3 bya (billion years ago), after the formation of oceans (~4.4 bya) and Earth (4.5 bya) [1,2]. Based on chemical evolution [3,4,5], life evolved on Earth without spontaneous generations, i.e., only those reactions could have taken place that was favorable under given conditions. One of the first abiotic chemical steps included the formose reaction catalyzed by organic bases (e.g., diethylaminoethanol) [6]. The formose reaction is actually a reaction network where glycolaldehyde and glyceraldehydes appear to be autocatalytic due to their formation from formaldehyde as well as retro-aldol reactions in water [7]. Recently, a simplified reaction pathway involving free radicals has been identified through density-functional theory (DFT) computational calculations leading to the formation of ribosyl and ribonucleosides, where catalysis by Ca^2+^ and CaOH^+^ was involved. These results suggest that Ca^2+^ cations may not be directly involved in the formation of ribose from glyceraldehyde [8]. Intermediary steps of the formose reaction involved aldol condensations, aldose-ketose isomerizations producing glycolaldehyde, glyceraldehyde, dihydroxyacetone, tetrose, pentose, and hexose. In agreement with Omran et al. [9], the study of the formose reaction, under alkaline conditions and moderate hydrothermal temperatures, should not solely focus on sugars, particularly ribose as part of the genetic material, but also on the origins of metabolism via other metabolic molecules. An increasing number of scientists think that the generation of the genetic material developed from the formose reaction to ribose and RNA [8,9,10,11,12,13,14,15,16]. However, the consecutive chemical reactions of this pathway are not known. The major goal of this special edition is to describe that reaction that started with abiotic reactions from ribose formation in the formose reaction, leading to preRNA and finally to genetic RNA. Here we deal with the first nucleic acid, termed preRNA, as a non-genetic molecule, to be replaced by genetic RNA only later. Genetic RNA (genRNA) is a complex molecule of high molecular weight functioning as a carrier of genetic codes in the RNA world and ever since in some viruses with each mRNA being itself a genetic code. To avoid misunderstanding the genetic code is a set of rules, an instruction, like a key, that allows us to translate information. A code is not the information itself but instruction on how to execute information transfer. In biology, we have a universal code, which tells the cells how to translate mRNA triplets (codons) into and amino acids. In the recent DNA Empire World each mRNA may transmit different genetic information, stemming from DNA transcription (could be different in RNA World) but each of these different genetic pieces of information is translated into the corresponding proteins by using the same and universal genetic code [17].

Processes generating life on Earth consisted of three successive stages: (a) abiotic synthesis of organic molecules; (b) the formation of molecular aggregates performing primitive metabolism (proto-metabolism), and (c) evolution into cells and organisms resembling those that exist today [3]. Ribose is the exclusive sugar component of nucleotides that could have reacted to form nucleotides of genetic importance. Many other nucleic acids (XNAs) could have reacted to form nucleotides of genetic importance [18]. The earliest prebiotic reactions described how biomolecules such as ribose, was synthesized and protected from degradation, and how nucleobases were generated from HCN oligomers and other derivatives [19,20,21,22,23]. 

Under prebiotic conditions, the cyanide ion on Earth was not toxic and became poisonous only when the oxygen atmosphere developed and iron-porphyrin species appeared [24]. Prebiotic conditions remained a subject of debate, but a reasonable aspect to consider is that structure shaping biomolecules consisted almost exclusively of elements belonging to the CHNOPS group, where capital letters correspond to the elements of carbon, hydrogen, nitrogen, oxygen, phosphorus and sulfur. A notable exception of bioelements could have been molecular oxygen that was absent in the atmosphere. This does not mean that oxygen-containing molecules such as CO and water were not present. Water was photolyzed and generated in statu nascendi hydrogen and oxygen in the atmosphere [25] containing N_2_, CO, during the Hadean geologic eon of the Earth about 4.6 billion years ago before the Archean. Hadean produced a variety of other small organic compounds, including C1−C3 hydrocarbons, alcohols, aldehydes, acetone, and acetic acid [26,27]. PreRNA precursors such as ribose and nucleobases came into being some 4.3–4.5 billion years ago. The initial reducing atmosphere existed much before the Great Oxygenation Event took place some 2.3–2.4 billion years ago [28]. After the primordial photolyzing period subsidized, the atmosphere became relatively unproductive again [29], much earlier than the Great Oxygenation Event occurred.

The resemblance between the periodicities of the chemical elements in the mendelian periodic table shows that bioelements belong to the 1–3 periods and contain only *s* and *p* electrons. Trace amounts of heavy metals, mostly in period four are required for specific biological processes. Several important roles for heavy metals have been published, but none of them have been widely accepted. The electronic configuration and the biological effect of elements served as reasonable orientation points to define heavy metals of biological importance [30]. At 3 g/cm^3^ and higher density values transition or *d-*metals have all roughly the same size and number of possible oxidation states causing significant incompatibility with bioelements. Based on these two criteria, heavy metals have been defined as those elements that have ≥3 g/cm^3^ densities and *d-* or higher electrons in their electronic configuration [5,30].

The spatial distribution of electrons reflects an axial symmetry of single bond formation between atoms and the tetrahedral geometry of *s-p* and *p-p* bond formations [29]. The tetrahedral bond angle of 109.5° is characteristic of elements containing *p* electrons belonging to the second and third-period elements.

Due to the bond valence of oxygen (2) and nitrogen (3) under that of carbon (4), the bond angles in water (104.5**°**) and ammonia (107.3°) make these molecules polar and somewhat distorted relative to the ideal tetrahedral bond angle as in methane (109.5°). The departure of an ideally tetrahedral angle of water, ammonia, ethers, and amines is due to the diminished symmetry around the O and N atoms. The angles of a methylene group, tertiary or quaternary carbon, bearing a central C atom of the same electronegativity as in methane, will also depart from the ideal tetrahedral angle, simply for asymmetry reasons. Water, ammonia, and other related compounds are polar because of the tetragonal arrangement of the electrons of the central atom, including the non-bonding electron-pairs, which results in close to tetrahedral H-X-H bond angles and non-zero overall molecular dipoles.

This work focuses on those reactions of abiotic synthesis of organic molecules with particular attention to ribose selected as the precursor to life [18] that made possible the formation of random–sequence non-coding RNA and further the development of genetic RNA. The probability of reactions does not mean certainty, nevertheless, it does not disclose compounds from the race that could have been involved in the origin of life. Only those compounds and reactions deserve consideration that has already been suggested and known for their evolutionary involvement.

## 2. Prebiotic Synthesis of Ribose

The oligomerization of formaldehyde is the traditional way to carbohydrate synthesis through the formose reaction resulting in a mixture of sugars of aldoses and ketoses of different chain lengths from trioses to hexoses [31,32,33,34]. The formose reaction is regarded as an autocatalytic self-condensation cycle [35] being autocatalytic in the formation of C2 and C3 sugars of glycolaldehyde and glyceraldehydes due to retroaldol reactions, that follow the initial synthesis from formaldehyde and, respectively glyceraldehyde, thus enhancing the stoichiometry of their formation. The formose reaction starts with the dimerization of formaldehyde followed by C2 to C6 saccharide formation. The origin of life is traced back to pentoses generated from mixtures of formaldehyde (C1), glyceraldehyde (C2), glyceraldehyde (C3), dihydroxyaceton (C3), aldoses (C2–C6), and ketoses (C2–C4) as well as minerals derived from boric acid (B(OH)_3_)). The formose reaction creates a mixture of sugars with varying sizes and shapes, of which ribose makes up less than 1%. Albert Eschenmoser’s group could significantly increase the yield of pentoses from the formose reaction by starting with formaldehyde and glycerolphosphate, which delivered up to 23% racemic ribose-2,4-diphosphate [36]. Thus Eschenmoser has proposed an alternative “glyoxylate scenario”, where glyoxylate [37] and its formal dimer, dihydroxyfumarate, were intended to be the central starting materials of a chemical constitution of primordial metabolism. These compounds served as sources for the formation of biogenic molecules such as sugars, including ribose. The selective formation of ketoses in the “glyoxylate scenario”, stands in stark contrast to the formose reaction, where a complex mixture of linear and branched aldoses and ketoses are produced and could constitute a pathway for the formation of carbohydrates. The two views of ribose formation advocate opposite sides of a research controversy. The reason why the formose reaction is favored is given below.

**Figure 1:**
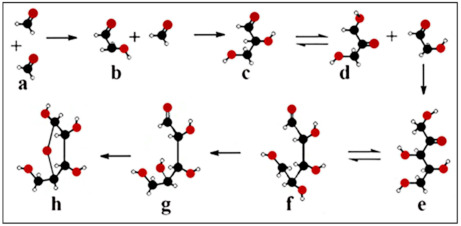
β-d-ribose synthesis in the formose reaction**.** Two formaldehyde molecules (**a**) condensed to glycolaldehyde (**b**). The subsequent aldol condensation is forming glyceraldehyde (**c**), which undergoes a ketose-aldose isomerization (**d**). Glyceraldehyde reacts with glycolaldehyde to bring about pentulose (**e**). Selective isomerization takes place between pentulose (**e**) and d-ribose (**f**), favoring the nucleophilic addition reaction of d-ribose (**g**) and ring formation to β-d-ribose (**h**).

Although, in the formose reaction at lower temperatures hexoses were synthesized in negligible yields but at a higher temperature (~200 °C) among pentoses ribose and ketoses resulted in a much higher proportion [29,34]. The missing selectivity for specific monosaccharide formation was one of the major problems of the formose reaction in the context of the origin of life [33,38,39]. The formose reaction encapsulated inside vesicles produced pentoses in 65% yield, which is much higher than the synthesis of C-5-monosaccharides under conventional conditions [40]. The same authors described that the system inside the vesicles as lipid-bound protometabolic units synthesizes complex carbohydrates and represents a fascinating example of an artificial cell capable of communication with natural cells [36]. Since ribose can be further transformed into flexible nucleotides where the intramolecular free rotation of functional groups is allowed and nucleotides can be polymerized to a genetic molecule [18] selective reactions favor the formose origin of ribose. Figure 1 shows ribose synthesis through the formose reaction.

It deserves to be mentioned in this context that the formose reaction does not deliver d-ribose enantioselectively, but only racemic ribose. d-ribose depicted here (for clarity purposes) is the natural enantiomer of biotic origin.

**Figure 2:**
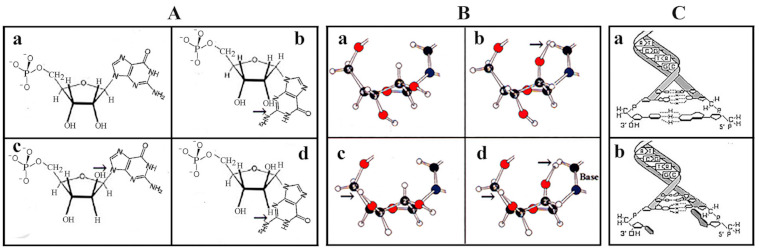
Selection of β-d-ribose as the best fitting pentose in the nucleotide structure. Irrespective of the formose or glyoxylate origin of ribose and the improved production of pentoses did not answer the question of why pentoses, particularly ribose, were selected as the exclusive sugar component of the genetic material. Molecular modeling revealed that free rotation of functional groups (OH, phosphate, nucleobase) of ribose inside nucleotides takes place only in β-d-ribose. (**A**/**a**), confirmed in (**B**/**a**). The free rotation of functional groups of the beta anomer of arabinose is hindered by its 2-OH moiety (**A**/**c**). As far as the alpha anomers of pentoses are concerned, the bulky bases are too close to the sugar and would not allow its free rotation (**A**/**b**,**d**). (**B**) confirms that free rotation of the substituents is permitted only in β-d-ribose (**B**/**a**) but not in arabinose, xylose, or lyxose (**B**/**b**,**c**,**d**). The free movement of functional groups of ribose turned out to be essential to provide maximal flexibility of RNA and to secure the stability and base pairing of deoxyribose in double-stranded DNA (**C**). Double helix formation is possible only in β-d-deoxyribonucleotides (**C**/**a**) but not between the α-d-deoxyribonucleotide pairs where the nucleobases are not perpendicular to the axis of the helix and will prevent hydrogen bonding between bases (**C**/**b**). With permission [18].

In nucleotides containing arabinose, xylose, or lyxose, the C2-OH or the C3-OH and the C5-OH groups are in the opposite orientation of the furanose ring substituents causing steric interference caused by the vicinity of the large base and/or the C5-OH group (Figure 1e). After ring formation in β-d-ribose all substituents are as far from each other as possible. The juxtaposed position of substituents (Figure 1h) provides free rotation of the substituents of β-d-ribose and high stability to the molecule. The conformers originating from sugar pucker folding as well as pentose configurations indicated that the selection of ribose was not a random process, but the only possible solution. β-d-ribose perfectly fits into functional nucleic acids, whereas other pentoses cause steric hindrances [41]. Thus for the formation of a flexible RNA chain, only β-d-ribose turned out to be suitable (Figure 2).

It should be also noted in this context that modeling with d-pentoses could also be modeled with l-pentoses and would yield identical energies and shapes, if not enantiomeric, provided that no mixture of d and l enantiomers were permitted [42]. However, the mirror image l-ribose is not found in nature [41]. 

## 3. Prebiotic Synthesis of Purine and Pyrimidine Nucleobases

The prebiotic synthesis of purine nucleobases under primitive Earth conditions [43] and the prebiotic pyrimidine synthesis started from cyanoacethylene (HC≡CCN) and cyanate (NCO^−^) [44]. Cyanyl (NCO) does not exist except under extreme vacuum conditions or in the cosmos, since it is a radical. The neutral, moderately stable compound is isocyanic acid (HNCO) [45]. Isocyanic acid forms upon strong heating of urea that eliminates ammonia to give isocyanic acid as a highly reactive liquid or gas. Ammonia and isocyanic acid can produce a quite volatile salt, ammonium cyanate. Cyanate as a sodium, potassium (or other metal cation) salt is more stable, but much less reactive, too. The chemical stability of the nitrogen-containing heterocyclic nucleobases suggests that they were among the first stable prebiotic molecules that have been formed by using HCN as the primary precursor. Less attention is paid here to the synthesis of nucleobases as it does not belong to the major focus of this study.

## 4. Phosphorylation, Polymerization, and Nucleotide Formation

Phosphate is a key component of life. This inorganic compound is a ubiquitous part of cellular components including nucleic acids backbone, the energy currency in small molecules (ATP), carrier of reduction potential (NADPH), and information (nucleotides). This ion made its transition from inert, mostly insoluble inorganic compounds into RNA and other biomolecules. Its transfer from inorganic salts into nucleic acids or other biomolecules takes place through a condensation reaction, by the removal of a water molecule. In nature, d-ribose is phosphorylated to d-ribose-1-monophosphate and d-ribose-5-monophosphate [46]. The mirror image l-ribose is not found in nature [47]. The transfer from inorganic salts into nucleic acids or other biomolecules involves a condensation reaction, by the removal of a water molecule. In the presence of water as a solvent in huge excess, the opposite reaction, the hydrolysis of the phosphor ester bond, takes place [40]. In naturally occurring biomolecules, d-ribose is present in the predominating β-d-ribofuranose form [48].

**Figure 3:**
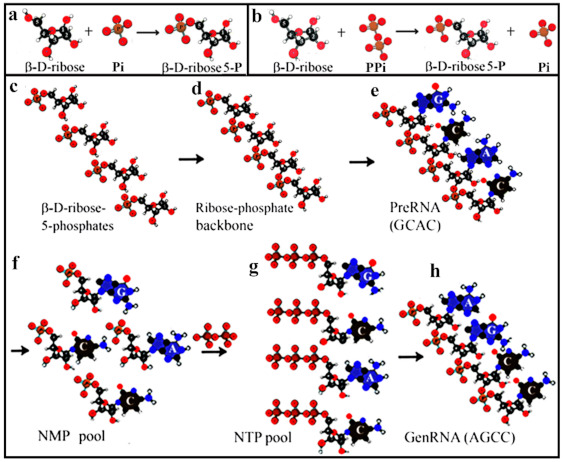
Selection of β-d-ribose as the best fitting pentose in the nucleotide structure. The two generally known phosphorylations of β-d-ribose are (**a**) phosphorylation with inorganic phosphate [39], (**b**) regioselective phosphate transfer from the activated pyrophosphate complex [40]. Before abiotic RNA generation is dealt with, it should be pointed out repeatedly that prebiotic reaction conditions, such as the formose reaction, can at best produce racemic ribosephosphate and other racemic compounds but no enantiomerically pure d-ribofuranose derivatives. Prebiotic RNA formation: (**c**) accumulation of β-d-ribose, (**d**) Polymerization of the sugar-phosphate backbone from β-d-ribose 5-phosphate units, (**e**) N-glycosidic bond formation between nucleobases and hydroxyl groups of the sugar-phosphate skeleton to bring about a random base, non-genetic preRNA. (**f**) Hydrolytic cleavage of nucleoside monophosphates and catalytic oligomers (not shown) from prebiotic RNA. (**g**) Phosphorylation with pyrophosphate (PPi) to NTPs. (**h**) NTPs serving as a pool for the synthesis of genetic RNA. Abbreviations: G, guanine; A, adenine; C, cytosine. Modified with permission [17].

Melvin Calvin found that dicyanamide promoted the formation of ribose 5-phosphate from ribose and orthophosphate [49] (Figure 3a). The phosphorylation of d-ribose using cyanogen and cyanamide as condensing agents resulted in β-d-ribofuranose 1-phosphate as the only sugar phosphate (10–20%) formed in the reaction of ribose with orthophosphate [50]. Recently Hu et al. presented a rapid, efficient, and regioselective phosphorylation method at the 5′-position of unprotected ribose and ribonucleosides with pyrophosphate in the gas phase [51] (Figure 3b). Ribose 1-phosphate to ribose-5-phosphate and vice versa could not be converted by proteinaceous catalysis as enzymatic reactions presumably did not exist at the time in prebiotic syntheses.

## 5. From preRNA to genRNA Formation

All prebiotic accumulation of ribose could be only racemic ribose. β-d-ribose 5-phosphates could have been polymerized through their C2′- or C3′-OH groups. Preferentially, the 3′-OH group of ribose serves the head-to-tail elongation and phosphodiester bond formation between two riboses (Figure 3c). To the ribose-phosphate backbone purine and pyrimidine bases were attached randomly bringing about preRNA, which was not a genetic molecule. The presence of the 2′-OH groups catalyzed the hydrolysis of preRNA to nucleoside monophosphates (NMPs) and oligomers (Figure 3d). The 2′-OH moiety is missing in deoxyribose and stabilizes DNA structure. NMPs were phosphorylated to NTPs (Figure 3e). The selection of NTPs took place and served as substrates for the selective genRNA synthesis (Figure 3f). As an example, the change from the random preRNA sequence (ACGC) to the genRNA primer sequence (AGCC) of *B. subtilis* is given [52]. It remains to be decided whether the first RNA contained all four bases found in contemporary RNAs [53].

## 6. Protection of Ribose

It was hypothesized that the stability of organic compounds assembled into polymers generally exceeded the stability of the same compounds as free monomers [54]. Equally important was the recognition that reactions providing higher stability stimulated the accumulation of prebiotic macromolecules especially among those that arose through the rearrangement of prebiotic phosphodiester polymers. Sugars joined by phosphodiester linkages underwent spontaneous transesterification reactions with selection for stability [54]. Neighboring prebiotic ribose phosphates could form phosphodiester bonds and polymerize into a sugar-phosphate backbone [55] suggesting that RNA can be synthesized not only by the polymerization of nucleotides (NTPs). This alternative involved (i) the abiotic formation of preRNAs, as non-genetic molecules that do not contain sequentially arranged information and (ii) hydrolysis of preRNA to catalytic oligonucleotides and nucleoside monophosphates (NMPs), the phosphorylation of NMPs to NTPs resulting in NTP substrates for the synthesis of genetic RNA [18].

The following recurring motifs in the chemical evolution deserve attention: (i) those spontaneous reactions that took place under conditions were the most favorable; (ii) only those products could be used as bridgeheads for the next step in evolution the stability of which has been secured. The formose reaction is generally accepted to produce numerous sugars including the prebiotic synthesis of ribose without any selectivity. Even if there were a selective synthesis of ribose, there would still exist the problem of stability. Sugars are known to be unstable in strong acid or base, but there are little data to store ribose under neutral solutions [56]. The stability of polyols is provided by joining them through phosphodiester linkages, undergoing spontaneous transesterification reactions with selection for stability. Selective stability of ribose was reported by borate [13], by silicate minerals [57]. Phosphate is specifically adapted for its role to protect nucleic acids because by connecting two ribose diesters against hydrolysis and still ionizes resulting in negative charge and stabilizes phosphomonoesters even more [58].

An important aspect of selecting ribose as the sugar components of RNA was that only this pentose could have been incorporated and processed further with higher frequency in the first metabolic route named formose–ribose–RNA pathway to form preRNA. As an analogy, the example of glyceraldehyde 3-phosphate (GAP) and dihydroxyacetone phosphate (DAP) is taken. In tissues the GAP/DAP ratio is about 1/20, nevertheless, GAP is processed further. One can argue that in glycolysis the isomerization of GAP <=> DAP takes place by the enzyme triose phosphate isomerase. However, catalysis is an ancient process that took place in the formose reaction. 

This is not the only generally accepted pathway bearing the burden of being favorable only under basic conditions in the presence of divalent metal cations.

The alternative that works at close neutral pH and even slightly acidic conditions, namely, through the photoreductive homologation of plausible hydrogen cyanide in the presence of sulfurous compounds (dihydrogen/monohydrogen sulfide, hydrogensulfide, thiophosphate) of volcanic provenance and catalyzed by ferrocyanide complexes as currently discussed by Menor-Salvan [59] and Sutherland’s group [20,60]. 

### 6.1. Catalysis in the Formose Reaction

It is assumed that the first stage of the origin of life began with the onset of nonenzymatic reactions catalyzed by naturally occurring metal ions and stabilized by minerals already mentioned. The catalytic formation of the very first monosaccharides enabled the appearance of “golden organic products” that contributed to the evolution of monosaccharides [33]. The formose reaction turns to an autocatalytic cycle for the formation of glycolaldehyde and glyceraldehyde in the presence of Ca^2+^ and CaOH^+^ cations [8]. 

This is due to retroaldol reactions, that follow their initial synthesis from formaldehyde and, respectively, glycolaldehyde, thus enhancing the stoichiometry of their formation. Hence, neither glycolaldehyde, nor glyceraldehydes actually catalyze their own formation in the classical mechanistic sense, but the stoichiometry of their formation is significantly enhanced by the chemical reaction network once it has started to be truly operative, which simulates the behavior of a truly autocatalytic cycle [61]. 

### 6.2. Formose–Ribose–RNA Pathway

**Figure 4:**
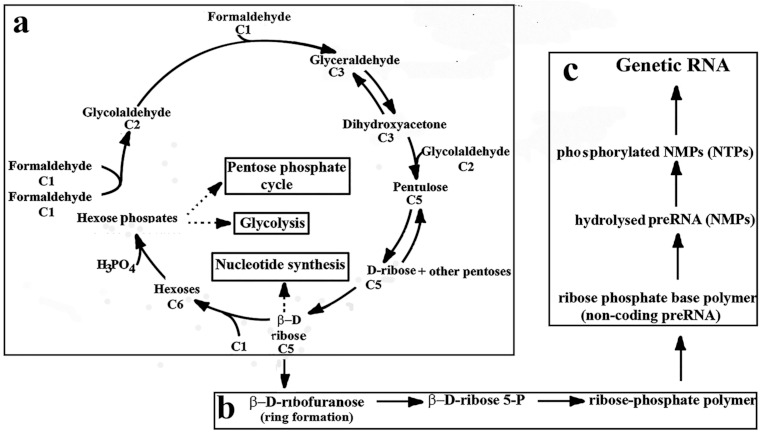
Theoretical **f**ormose–ribose–RNA pathway. The pathway consists of three parts. (**a**): Formose reaction: Two formaldehyde (C1) forms glycolaldehyde (C2). Formaldehyde (C1) and glycolaldehyde (C2) react to give glyceraldehyde (C3). Glyceraldehyde isomerizes reversibly to dihydroxyacetone (C3). The addition of glycolaldehyde (C2) to dihydroxyacetone (C3) generates pentulose (C5) that isomerizes to d-ribose and other pentoses (C5). Insets: The formose reaction is the generator of abiotic metabolism and thought to initiate other pathways such as pentose-phosphate pathway, glycolysis, nucleotide biosynthesis, (**b**): Polymerization of β-d-ribose preceded by ring formation to d-ribose, the phosphorylation of β-d-ribose, and polymerization of β-d-ribose-5P to form the ribose-phosphate backbone. (**c**): Attachment of nucleobases to the ribose-phosphate backbone to bring about preRNA, hydrolysis of preRNA to the smallest nucleotide units (NMPs), the phosphorylation of which leads to NTPs, the substrates of genRNA. Double arrows show the reversibility of the reactions. Dotted arrows refer to processes developed later during the evolution.

Parts of the pathway were described in Figure 1 and Figure 3. Here we join the consecutive steps of these parts into the Formose–Ribose–RNA pathway (Figure 4). 

## 7. Hierarchical Processes in the Transfer of Cellular Information

The RNA world hypothesis suggested that life started with single self-replicating molecules. Among several other hypotheses, autocatalytic sets could have been formed that used initially metals and other small self-produced molecules as catalysts that were able to produce basic blocks first for RNA, then for proteins. Without committing to hypotheses, the author favors the abiotic reactions that were likely to occur at the beginning of evolution and led to RNA synthesis and the formation of life. Unlike nucleic acids, proteins could not serve as templates for their own synthesis, a highly complex apparatus must have existed before proteins could have been made, and this apparatus consisted mainly of RNA. The other argument in favor of the gene-first/RNA world hypothesis was much more obvious, stating that RNA was also the primeval genetic material [8,62]. The hypothesis supported the existence of the first crystal structures of the ribosome, which clearly showed that proteins [47], atypically small ribosomal proteins played only supporting roles in the transmission of genetic information [63]. A different scenario is based on autocatalytic sets of molecules that were able to produce sufficient basic building blocks for RNA. After these larger molecules came into being, they could have taken over the role of original catalysts [7]. Alternatively, RNA aptamers are supposed to control gene expression in response to small molecules in mammalian cells. These riboswitches offer attractive features such as small genetic size and lower risk compared to protein-based transcriptional gene switches [64]. The claims that autocatalytic sets of organic polymer molecules could undergo evolution by themselves need further confirmation [65].

**Figure 5:**
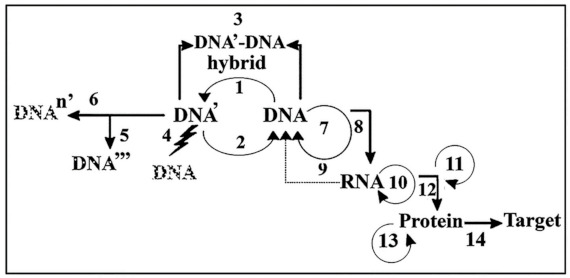
The hierarchical arrangement of transfer processes involved in genetic information. Processes of the DNA Empire take place at DNA, RNA and protein levels: **DNA↔DNA transfer processes**: 1. Mutation: DNA => DNA’; 2. DNA repair: DNA’ => DNA; 3. Recombination: crossover, gene conversion (DNA’–DNA hybridization). Recombination can take place intracellularly but is mainly an intercellular process; 4. Apoptosis (programmed cell death, high levels of DNA damage); 5. Aging, several mutations, persistent DNA damage: DNA’ => DNA’’ => DNA’’’; 6. Malignant transformation with multiple mutations: DNA => DNA^n^’ (persistent DNA damage, many mutations, mutant p53); 7. DNA replication: DNA <=> high fidelity (HiFi) process, (1:10^10^ misincorporated deoxyribonucleotide). **DNA↔RNA processes**: 8. Medium-fidelity (MeFi) process, 1:10^5^ misincorporated ribonucleotide); 9. Reverse transcription: RNA => DNA (in retroviruses); 10. RNA replication: RNA <=> RNA. **Processes belonging to posttranscriptional modifications**: 11. 5’-cap formation, 3’-polyA formation, splicing; 12. Translation: RNA => protein (low fidelity, LoFi process, 1:10^4^ misincorporated amino acid). **Processes belonging to protein modification**: 13. protein splicing, transglutamination; 14. Protein targeting: information reaches its intra- or extra-cellular destination. Figure 5 was used with the permission of [22]. Dotted letters of DNA represent mutations. Arrow resembling lightning refer to many mutations occurring within a short period of time.

The recognition of the hypothesis that among the genetic molecules, RNA came first, protein second, and DNA only third suggests that RNA and probably an even earlier ancestor molecule, namely ribose could be the first molecule that was related to and initiated the genetic system. Processes belonging to the intracellular transfer of genetic information are summarized in Figure 5. 

## 8. Conclusions

As far as the genetic origin of life on Earth is concerned, evolution selected among those chemical solutions that, a) generated catalytic networks, (b) contained chemical sequence information, (c) produced a reproducible and stable living system, and (d) adapted to the gradually changing environmental conditions (temperature, pressure, irradiation flux, availability as well as the concentration of feedstock molecules). The formose reaction deserves special attention since it allows the formation of many compounds, among them, sugars. However, hexoses and the majority of pentoses posed evolutionary barriers, and only those reactions could polymerize into a flexible genetic molecule that contained β-d-ribose. The skeleton of a hypothetical preRNA could have evolved through strong covalent bonds containing different bond energies and bond dissociation energies. Evolution selected a crystal lattice of flexibly attached complimentary copy that could be quickly reproduced. The prebiotic synthesis of RNA followed the order of the strength of bonded atoms, namely the stronger the bond, the higher its energy, and shorter its length. Correspondingly, the chemical interactions and bond formations reflect a priority order from the stronger bonds to the weaker ones starting with: short covalent > longer covalent bonds > van der Waals interactions > ionic bonds > hydrogen bonds.

Short but strong covalent bonds were formed between the 3′C-OH and 5′-phosphate groups of consecutive riboses through two ester bonds in the sugar-phosphate backbone. The N-glycosidic bonds represent longer but weaker covalent interactions. The reason why the phosphodiester linkages could be involved in preRNA resulting in nucleotides (NMPs) is related to the presence of their reactive 2′C-hydroxyl groups. The 2′C-OH absent in DNA contributes to the stability of the phosphodiester linkage even under alkaline hydrolysis. The presence of reactive 2′-OH in preRNA gives a reasonable explanation of why the ribonucleoside monophosphate chain of preRNA could have been hydrolyzed, although ribonucleotides were not present during preRNA formation. It is concluded that the abiotic route of RNA synthesis started with the utilization of d-ribose.

The formose reaction generated small amounts of racemic ribose and continued and continued through the polymerization of ribose 5-phosphate then by the steps of the non-genetic and genetic RNA synthesis. It is assumed that the very first steps of abiotic chemistry started with the spontaneous formation of simple compounds dictated by several factors. Initial reactions followed the order of bond strength, the availability, and concentration of substrates and catalysts. Only those chemical changes were able to develop into a chain of metabolic reactions where the stability of intermediates could be secured and conformed to the conditions prevailing on Earth. Occasionally the next step in evolution could be predicted, or at least guessed. The compounds of such reactions are referred to as the molecules of providence. Among these molecules, the most important ones are the informational macromolecules, RNA, DNA, and proteins. However, RNA would not have been created without ribose, more precisely, ribose 5-P, thus ribose-5-phosphate also belongs to the category of molecules of providence. Molecules of providence impact the direction of evolution, refer to predictable chemical processes, and an acceptable term of providence for both religious and non-religious people.

## 9. Brief Summary

Molecular models served as valuable teaching aids. By means of our models (>40) several questions related to pentose and hexose formation, nucleic acid structure, structure, flexibility, coiling, and supercoiling could be answered. Models helped to clarify why the free rotation of OH, phosphates, and nucleobase substituents of pentoses selected ribose as the exclusive sugar component of nucleic acids. Those potential abiotic reactions have been lined up and developed have developed into a pathway that could have resulted in prebiotic ribose formation, to the protection of ribose by phosphorylation, polymerization, attachment of nucleobases to the ribose-phosphate backbone resulting in abiotic RNA. The hydrolysis of preRNA released NMPs that by phosphorylation to NDPs and NTPs, then served as substrates for the genetic RNA synthesis.

## Data Availability

MDPI Research Data Policies at https://www.mdpi.com/ethics (accessed on 7 April 2021).
